# Colorimetry-Based Detection of Nitric Oxide from Exhaled Breath for Quantification of Oxidative Stress in Human Body

**DOI:** 10.3390/healthcare9081055

**Published:** 2021-08-17

**Authors:** Muni Raj Maurya, Haseena Onthath, Hagar Morsy, Najam-US-Sahar Riyaz, Muna Ibrahim, Alaa Elsafi Ahmed, Raghad Abuznad, Aeshah Alruwaili, Fatimatulzahraa Alsaedi, Peter Kasak, Kishor Kumar Sadasivuni

**Affiliations:** 1 Center for Advanced Materials, Qatar University, Doha 2713, Qatar; muni88_raj@yahoo.co.in (M.R.M.); haseena@qu.edu.qa (H.O.); hm1604839@qu.edu.qa (H.M.); nr1703094@student.qu.edu.qa (N.-U.-S.R.); mi1701758@qu.edu.qa (M.I.); aa1702336@qu.edu.qa (A.E.A.); muni.raj@qu.edu.qa (R.A.); mh1208170@student.qu.edu.qa (A.A.); Fa1704598@qu.edu.qa (F.A.); peter.kasak@qu.edu.qa (P.K.); 2 College of Arts and Science, Qatar University, Doha 2713, Qatar

**Keywords:** colorimetry, m-Cresol Purple, Nitric oxide, exhaled breath, biosensor

## Abstract

Monitoring exhaled breath is a safe, noninvasive method for determining the health status of the human body. Most of the components in our exhaled breath can act as health biomarkers, and they help in providing information about various diseases. Nitric oxide (NO) is one such important biomarker in exhaled breath that indicates oxidative stress in our body. This work presents a simple and noninvasive quantitative analysis approach for detecting NO from exhaled breath. The sensing is based on the colorimetric assisted detection of NO by m-Cresol Purple, Bromophenol Blue, and Alizaringelb dye. The sensing performance of the dye was analyzed by ultraviolet–visible (UV–Vis) spectroscopy. The study covers various sampling conditions like the pH effect, temperature effect, concentration effect, and selective nature of the dye. The m-Cresol Purple dye exhibited a high sensitivity towards NO with a detection limit of ~0.082 ppm in the linear range of 0.002–0.5 ppm. Moreover, the dye apprehended a high degree of selectivity towards other biocompounds present in the breath, and no possible interfering cross-reaction from these species was observed. The dye offered a high sensitivity, selectivity, fast response, and stability, which benchmark its potential for NO sensing. Further, m-Cresol Purple dye is suitable for NO sensing from the exhaled breath and can assist in quantifying oxidative stress levels in the body for the possible detection of COVID-19.

## 1. Introduction

In the 1980s, Nitric oxide (NO) was discovered as the first endogenously produced gaseous signaling molecule [1]. It is the only endogenous molecule that can act as a neurotransmitter, constructive mediator, inducible mediator, and cytotoxic molecule [2]. The free radical nature of NO is the reason behind its unusual properties and multiple functions [3]. Due to these various physiological roles, the imbalance of NO leads to more than a dozen pathophysiological conditions like hypertension, stroke, cardiac failure, CNS disorder, diabetes mellitus, and many others [4]. There are reports establishing the relation between an increased NO level and bronchial asthma [5,6]. Even the numerous metabolic paths of NO in respiratory pathophysiology have been studied [7]. NO can act as a messenger of various effects like nonadrenergic, noncholinergic neurotransmission, vascular and nonvascular smooth muscle relaxation, etc. [7]. The origin of NO is found in the airway epithelium [8], and its increased level can determine several lung diseases [9]. Thus, NO monitoring devices for diagnosing diseases by noninvasive methods like exhaled breath fingerprinting would be a benchmark [10]. Exhaled breath is a biological medium that carries relevant medical information. The human exhaled breath contains various kinds of VOCs (Volatile Organic Compounds) like NO, CO, CO_2_, NH_3_, isopropanol, acetone, etc. that serve as biomarkers for the diagnosis of different diseases [11,12,13]. They originate from normal and metabolic processes occurring in the body. Thus, investigating metabolites in exhaled breath brings out valuable information about malfunctions and subsequent diseases occurring in the human body. Nitric oxide plays a crucial role as an intra- and extracellular mediator in many physiological and pathological processes such as the modulation of vascular tone, thickening and remodeling of the bronchial muscle wall, and regulation of local inflammation [14]. As a result, NO concentration in the exhaled breath is widely studied for the diagnosis of different health conditions such as asthma [15], COPD (Chronic Obstructive Pulmonary Disease) [16], Hypertension, PAH (Pulmonary Arterial Hypertension), and significant conditions like lung diseases [14] and even Covid-19 [17], etc. The NO concentrations in exhaled breath that correspond to different health problems are listed in Table 1.

Recently, Covid-19 diagnosis has been reported by analyzing the VOCs in the exhaled breath using gas chromatography methods like mass spectrometry (GC-MS) and ion mobility spectrometry (GC-IMS) [18]. In humans, a transmembrane protein, angiotensin-converting enzyme 2 (ACE2), serves as the main entry point into cells for the coronavirus family and even COVID-19. ACE2 enzyme is attached to the endothelium and outer surface of cells present in the heart, lungs, intestines, and kidney [19]. The interaction of the coronavirus with ACE2 and endothelium damages their protocol of biological activities and creates an unbalanced metabolism of the cell, often regarded as symptoms of the disease. The prominent nonfunctioning of the endothelium results in the imbalance of reactive oxygen species production [19]. The primary oxygen compound that is greatly affected by the nonfunctioning of the endothelium is NO [19,20]. The imbalance of the NO proportion in the body is directly related to the cause of hypertension [20]. Even in the coronavirus-infected person, the chances of individuals suffering from hypertension are observed, and their death percentage is quite noticeable [20]. Thus, the effective quantification of the NO in the exhaled breath will help distinguish persons infected by the coronavirus and healthy persons.

Various sensing techniques have been reported to diagnose health conditions in accordance with different biomarkers. The most frequently used method for the detection of NO in exhaled breath is a chemiluminescence analyzer [21]. Apart from this, Berkeley NO Test Strips detect NO from saliva [22]. The other techniques used for NO measurements include, colorimetric, fluorometric, electron spin resonance (ESR), electrochemical, etc. [2]. For the real-time application of NO sensors, it is essential to exhibit sensitivity in the range of ppm levels [23,24]. Compared to the other analytical methods, the colorimetric assay has demonstrated great attentiveness due to salient features like simplicity, rapid execution, naked-eye detection, and sensitivity in detecting the analyte concentration in ppm levels. Thus, it is intriguing to study the possibility of measuring NO concentrations using colorimetry, which can lead to the development of noninvasive exhaled breath NO analyzers to diagnose diseases.

In this paper, we report simple dye-based colorimetry sensing to quantify NO levels. For accuracy and stability, the dyes sensing was analyzed at different pH levels and temperature ranges. Moreover, the sensor selectivity towards NO has been verified with respect to other biomarkers present in the exhaled breath.

## 2. Materials and Methods

### 2.1. Materials Used

Bromophenol Blue, m-Cresol Purple, and Alizaringelb dyes were purchased from E. Merck, Darmstadt, Germany. Nitric acid (HNO_3_) (69–72%) was obtained from Research-Lab Fine Chem Industries, Mumbai, India, and Cu wire (R/C 6.35 × 0.61) from Maksal, South Africa. The compounds used for the selectivity analysis are; Ammonia (NH_3_) (25% from Riedel-de-Haën Germany), Hydrogen peroxide (H_2_O_2_) (30% from Meiiorate Health Opc Pvt. Ltd. Mumbai, India), Benzene (C_6_H_6_) (99% BDH Ltd. Pool England), C_3_H_8_O (>99% from Fluka Chemie, Switzerland), Deionised (DI) water from Millipore Milli-Q water system. All reagents used for analysis were of analytical grade.

### 2.2. Experimental Methods

The stock solution of Bromophenol Blue, m-Cresol Purple, and Alizaringelb dyes were prepared by dissolving 6 mg of respective dye in 100 mL of DI water. The stock solution of dyes was further diluted three times, and the obtained diluted solution was used in all the experiments. NO gas was prepared by adding 10 g of Cu in 5N HNO_3_. The resultant reaction byproduct gas, i.e., NO, was bubbled directly into the enclosed bottle containing 200 mL of DI water. After passing the NO, its concentration in the water was estimated by titration with KMnO_4_. The as-obtained stock solution of NO was further diluted to prepare different concentrations of NO solution ranging from 0.002 ppm to 50 ppm.

### 2.3. Characterization

A Biochrom UV Spectrophotometer (scanning range; 190–1100 nm) with a scanning range of 300–750 nm was used for ultraviolet–visible (UV–Vis) spectroscopy.

## 3. Results

### 3.1. Colorimetry Response of Dye in Different pH

**Figure 1:**
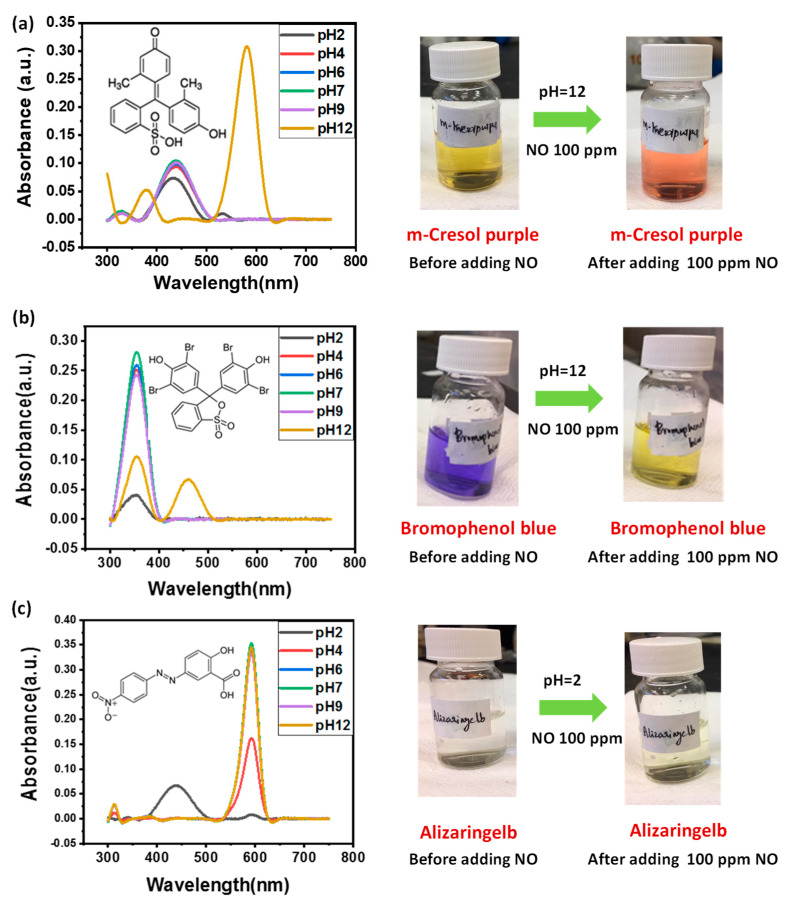
The effect of NO at different pH levels of dyes’ solutions. (**a**) UV–Vis spectra of m-Cresol Purple dye solutions after adding 100 ppm of NO (**left**) along with a photograph of the dye solution (**right**). (**b**) UV–Vis spectra of Bromophenol Blue dye solutions after adding 100 ppm of NO (**left**) along with a photograph of the dye solution (**right**). (**c**) UV–Vis spectra of Alizaringelb dye solutions after adding 100 ppm of NO (**left**) along with a photograph of the dye solution (**right**).

The m-Cresol Purple, Bromophenol Blue, and Alizaringelb dye solutions (10 mL) with pH 2, 4, 6, 7, 9, and 12 were prepared, and 1 mL of 100 ppm NO test solution was added at room temperature. The response time for the dyes was estimated by calculating the time from the addition of NO to the corresponding visible color change observed in the dyes’ solution. Figure 1a shows the m-Cresol Purple detection towards NO in different pH solutions. A significant visible color change was noticed in the pH 12 dye solution. The color change from yellow to orange in the presence of the 100 ppm NO test solution was confirmed by the emergence of a new absorption band centered at ~581 nm in the UV-Vis absorbance spectra, as shown in Figure 1a (left). Figure 1a (right) shows the photograph of the m-Cresol Purple dye solution before and after the addition of the 100 ppm NO test solution. The bare dye solution exhibited a yellow color, and a visible change to orange color was noticed after adding 100 ppm of NO. The estimated response time of m-Cresol Purple dye was ~2 s. For the NO detection analyses performed in Bromophenol Blue dye, a color change was noticed in the pH 12 solution, while no such color change was observed in the acidic and neutral medium. Figure 1b (left) shows the UV–Vis spectra of Bromophenol Blue dye solutions (pH 2–12) after adding 100 ppm of NO test solution. Compared to other pH solutions, an absorption band centered at ~460 nm is observed in UV–Vis spectra of the pH 12 Bromophenol Blue solution. The corresponding visible color change of the dye after adding 100 ppm on NO in pH 12 dye solution is shown in Figure 1b (right). From the figure, a visible color change from blue to yellow is clearly observed. The response time of Bromophenol Blue dye was estimated to be ~5 min. Further, for the NO assay in Alizaringelb solutions with different pH, no significant visible color change was noticed except in the pH 2 solution. The pH 2 solution of Alizaringelb dye turns slightly yellowish after adding 100 ppm of NO, and a corresponding color change is indicated by the emergence of an absorption band centered at ~440 nm in the UV–Vis spectra, as shown in Figure 1c (left). The photograph of the pH 2 Alizaringelb dye solution before and after adding 100 ppm NO is shown in Figure 1c (right). Moreover, the dye apprehended a response time of ~1 min.

Interestingly, compared to the Bromophenol Blue and Alizaringelb dyes’ solution, the NO assay in the m-Cresol Purple dye solution exhibited a noticeable visible color change with a fast response time of ~2 s. Thus, from the above investigation, it can be inferred that the m-Cresol Purple dye is best suited for the colorimetric-based sensing of NO. The observable color change offers a convenient approach to detect NO via unaided eyes. To further elaborate on the sensitivity of the m-Cresol Purple dye towards NO detection, a systematic analysis was carried out by testing different ppm levels of NO in the dye solution.

### 3.2. Sensitivity Analysis

**Figure 2:**
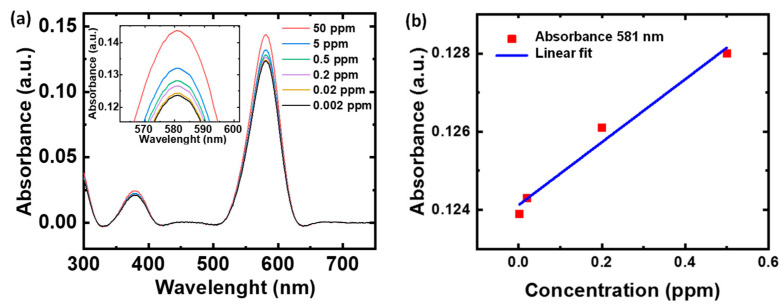
m-Cresol Purple dye sensitivity towards NO detection. (**a**) UV–Vis absorption curve of dye with change in NO concentration. Inset shows the magnified plot of the absorbance peak centered at 581 nm. (**b**) Corresponding calibration plot of dye in the detection range of 0.002–0.5 ppm.

To analyze the sensitivity of the dye towards NO detection, the UV-Vis study was carried out by varying the NO concentration from 0.002 to 50 ppm in the pH 12 m-Cresol Purple dye solution. Figure 2a shows the UV–Vis absorbance plot of the dye with a change in NO concentration from 0.002–50 ppm. For the absorption peak at 581 nm, an increase in absorbance is observed with an increase in the NO concentration (Figure 2a). The inset in the figure shows the magnified plot of the absorbance peak centered at 581 nm. The corresponding calibration plot for estimating the dye limit of detection (LOD) towards NO sensing is shown in Figure 2b. The calibration curve was plotted by considering the peak absorbance of dye at 581 nm for different concentrations of NO. Linear fitting was performed to estimate the LOD using Equation (1):

(1) $$\mathrm{LOD}=\;\frac{3\sigma }{m}$$

where m represents the slope of the calibration plot, and σ is the standard deviation of the intercept. Linear fitting was performed in the range of 0.002–0.5 ppm NO, and the estimated LOD was ~0.082 ppm, y = (0.00805 ± 0.8.17 × 10^−4^)x + (0.124 ± 2.2 × 10^−4^); R^2^ = 0.979. The sensitivity investigation indicates that the m-Cresol Purple dyes exhibit a high sensitivity towards NO with a linear detection limit as low as ~0.082 ppm in the concentration range of 0.002–0.5 ppm.

### 3.3. Temperature Effects

**Figure 3:**
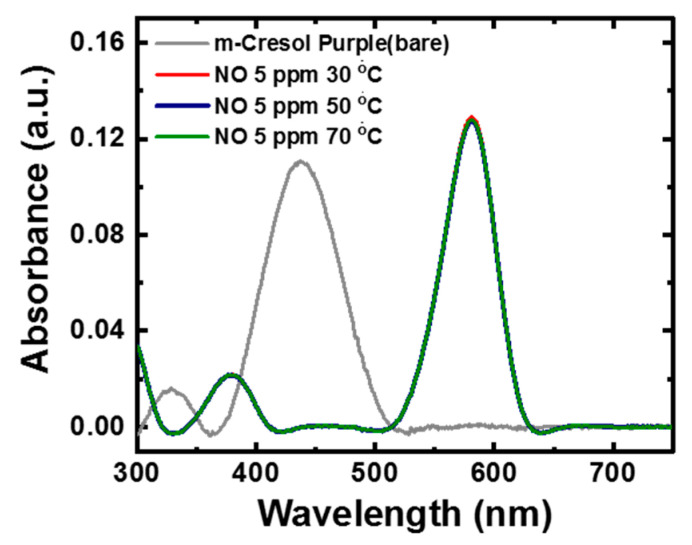
The UV–Vis absorbance plot of m-Cresol Purple dye after adding 5 ppm of NO at different temperatures.

One of the most important properties for sensing applications is the stability of a sensor toward the change in temperature of the surrounding or involved medium. Thus, to investigate the stability of the dye response with respect to change in temperature, the dye solution was subjected to different temperatures, and sensing towards NO was analyzed using UV–Vis absorbance spectroscopy. Figure 3 shows the effect of temperature on the sensing performance of m-Cresol Purple dye (pH 12) towards 5 ppm NO. It is noticeable that the absorbance curve of dye treated with temperatures in the range of 30 °C, 50 °C, and 70 °C exhibits a negligible deviation during NO detection. The temperature study reveals that there is no significant effect of temperature on dye sensing. The above investigation suggests that the colorimetry dye sensor offers a high stability towards the change in temperature, which is very important for getting the real-time NO sensing behavior from the exhaled breath.

### 3.4. Selectivity

**Figure 4:**
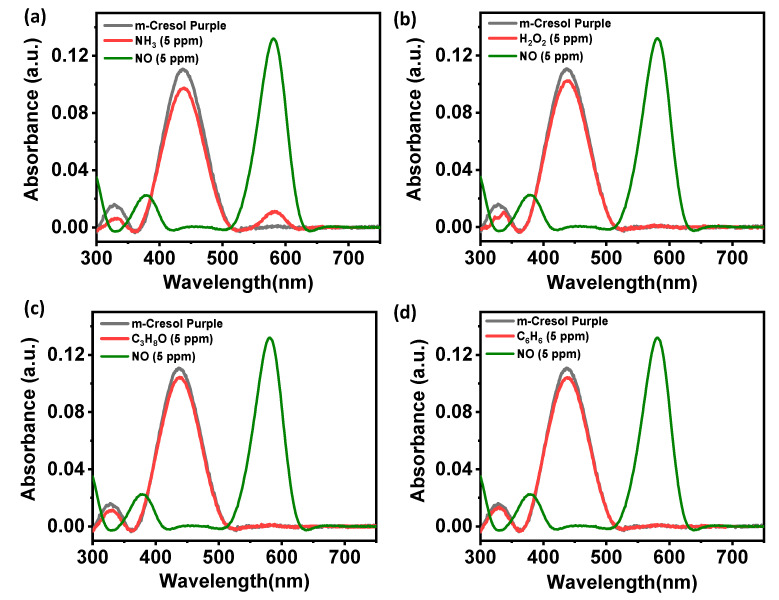
Selectivity analysis of m-Cresol Purple dye. (**a**) Ammonia. (**b**) Hydrogen peroxide. (**c**) Isopropyl alcohol. (**d**) Benzene.

In order to evaluate the selectivity of dye towards NO detection, control experiments were conducted in pH 12 m-Cresol Purple dye solution with potential interfering biomarkers in the breath like H_2_O_2_, C_6_H_6_, NH_3_, and C_3_H_8_O. The test solution concentration of NO and potential interfering analytes was 5 ppm. Figure 4 shows the comparison of UV–Vis spectra of bare dye solution, dye solution after adding NO, and different analytes. In the case of ammonia, a slight color change was noticed (see Figure 4a). However, the visible blue color was obtained after adding ammonia, which was very distinct from the visible orange color noticed after adding NO to the dye solution. On the other hand, no visible color change was noticed for H_2_O_2_, C_3_H_8_O, and C_6_H_6_. Moreover, after adding different analytes, the UV–Vis study reveals a negligible shift in the absorbance peak centered at ~439 nm, as shown in Figure 4a–d.

**Figure 5:**
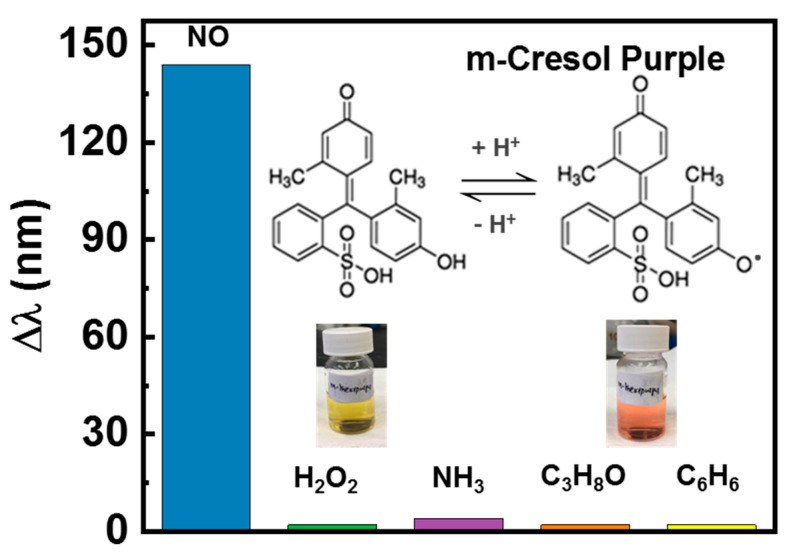
Selectivity analysis of m-Cresol Purple dye towards NO and other analytes.

Further, m-Cresol Purple dye selectivity for NO was estimated by measuring the shift in the maximum absorption wavelength (Δλ) obtained in the UV–Vis spectra. The peak shift factor is estimated by Equation (2):

(2) $$\Delta \lambda =\;\lambda -{\;\lambda }_{0}$$

where λ and λ_0_ are the wavelengths of the maximum absorbance measured in the presence of the test analyte and blank dye solution, respectively. The quantitative analysis of the dye selective nature towards NO detection is shown in Figure 5. Compared to other analytes, the addition of NO induces the peak shift that corresponds to the visible color change of m-Cresol Purple dye from yellow to orange. Thus, a significant peak shift is observed for NO compared to the other interfering compounds. The addition of the NO in m-Cresol Purple dye can result in a loss of proton from a phenolic hydroxyl group of the dye molecule, as shown in the inset of Figure 5. The induced loss of proton leads to the formation of a new compound, which is indicated by a dramatic visible color change of the dye solution from a yellow to orange color. These results clearly indicate that other interfering substances showed a negligible competition towards NO detection. Consequently, m-Cresol Purple dye is inert to NO detection and displays a potential towards being a highly selective colorimetric sensor for the NO molecule. As a result, m-Cresol Purple dye appears to be a promising candidate for the noninvasive colorimetry-based detection of NO levels in exhaled breath. It can further assist in quantifying oxidative stress levels in the body.

## 4. Conclusions

In summary, the systematic investigation of colorimetry sensing of m-Cresol Purple for NO sensing has been presented. The NO sensing performance of the dye was analyzed by the UV–Vis spectroscopy technique. From colorimetry studies, the dye catalyst exhibited a high sensitivity with a detection limit of ~0.082 ppm in the linear range of 0.002–0.5 ppm. The dye also exhibited a high stability with temperature change. The m-Cresol Purple dye shows an outstanding sensitivity, selectivity, fast response, and stability towards the colorimetry-based detection of NO. Moreover, the dye appears to be a promising candidate for the colorimetry-based detection of NO levels in exhaled breath. The noninvasive NO sensor can further be implemented to quantify oxidative stress levels in the body.

## Figures and Tables

**Table 1:** NO concentration in exhaled breath for different health conditions.

Disease/Condition	NO Level
Covid 19	≤15 ppb
Asthma	>45 ppb
Severe COPD	<10 ppb
PAH	<10 ppb
Heart failure	>20 ppb
Atherosclerosis	<10 ppb
Psoriasis	>20 ppb

## Data Availability

The data presented in this study are available on request from the corresponding author.
